# Chinese College Students Have Higher Anxiety in New Semester of Online Learning During COVID-19: A Machine Learning Approach

**DOI:** 10.3389/fpsyg.2020.587413

**Published:** 2020-12-03

**Authors:** Chongying Wang, Hong Zhao, Haoran Zhang

**Affiliations:** ^1^Department of Social Psychology, Zhou Enlai School of Government, Nankai University, Tianjin, China; ^2^Department of General Computer, College of Computer Science, Nankai University, Tianjin, China

**Keywords:** COVID-19, college students, anxiety, new semester, machine learning, XGBoost model

## Abstract

The COVID-19 pandemic has caused tremendous loss starting from early this year. This article aims to investigate the change of anxiety severity and prevalence among non-graduating undergraduate students in the new semester of online learning during COVID-19 in China and also to evaluate a machine learning model based on the XGBoost model. A total of 1172 non-graduating undergraduate students aged between 18 and 22 from 34 provincial-level administrative units and 260 cities in China were enrolled onto this study and asked to fill in a sociodemographic questionnaire and the Self-Rating Anxiety Scale (SAS) twice, respectively, during February 15 to 17, 2020, before the new semester started, and March 15 to 17, 2020, 1 month after the new semester based on online learning had started. SPSS 22.0 was used to conduct *t*-test and single factor analysis. XGBoost models were implemented to predict the anxiety level of students 1 month after the start of the new semester. There were 184 (15.7%, Mean = 58.45, SD = 7.81) and 221 (18.86%, Mean = 57.68, SD = 7.58) students who met the cut-off of 50 and were screened as positive for anxiety, respectively, in the two investigations. The mean SAS scores in the second test was significantly higher than those in the first test (*P* < 0.05). Significant differences were also found among all males, females, and students majoring in arts and sciences between the two studies (*P* < 0.05). The results also showed students from Hubei province, where most cases of COVID-19 were confirmed, had a higher percentage of participants meeting the cut-off of being anxious. This article applied machine learning to establish XGBoost models to successfully predict the anxiety level and changes of anxiety levels 4 weeks later based on the SAS scores of the students in the first test. It was concluded that, during COVID-19, Chinese non-graduating undergraduate students showed higher anxiety in the new semester based on online learning than before the new semester started. More students from Hubei province had a different level of anxiety than other provinces. Families, universities, and society as a whole should pay attention to the psychological health of non-graduating undergraduate students and take measures accordingly. It also confirmed that the XGBoost model had better prediction accuracy compared to the traditional multiple stepwise regression model on the anxiety status of university students.

## Introduction

COVID-19 has spread rapidly around the world and the number of people who were affected has increased dramatically since early 2020 (World Health Organization, 2020). The unprecedented swift and strict quarantine measures starting from late January in China have kept a huge number of people in isolation or socially distancing, and hence has influenced their mental health and psychological resilience (Brooks et al., 2020; Wang et al., 2020a; Xiang et al., 2020). Many studies have noted the psychological impact of COVID-19 such as post-traumatic stress symptoms, confusion, anger, helplessness, fear, depression, and anxiety, etc., in the general population during the initial phase of the COVID-19 outbreak in China (Chew et al., 2020; Li et al., 2020; Qiu et al., 2020; Wang et al., 2020a,b).

During school closures, college students were quarantined and attended their new semester remotely through online learning, and experienced different levels of psychological pressure (Cao et al., 2020; Wang and Zhao, 2020). Bruffaerts et al. (2018) found that university students were more vulnerable and easily affected by a pandemic. Previous studies have reported a higher level and prevalence of anxiety among college students during an epidemic (Jia et al., 2003; Chen et al., 2004; Li et al., 2011; Yang et al., 2015). Chang et al. (2020) investigated 3881 college students in Guangdong province in China during the epidemic of COVID-19 and found that 26.6% of students had different levels of anxiety (23.19% mild, 2.71% moderate, and 0.70% severe) and depression was detected in 21.16% of them. Cao et al. (2020) also reported that 24.9% of medical college students experienced different levels of anxiety during COVID-19. However, there was no research on how their psychological stress and anxiety changed during the outbreak.

There were many factors found to be related to anxiety, Wheaton et al. (2012) reported that health anxiety, contamination fears, and disgust sensitivity were significant predictors of swine flu-related anxiety during the H1N1 influenza pandemic of 2009–2010. Social distancing, worries about their own health and the health of their families, stay-at-home orders, and limited physical interaction with others all counted toward their anxiety and stress (Zuckerman, 1989; Martin, 2010; Cao et al., 2020). Faramarzi et al. (2014) demonstrated that moral intelligence and identity status both contributed to the mental health problems of healthcare students. They used regression analysis or a stepwise model of multiple regression analysis to assess the correlation between different variables to investigate psychological characteristics. However, the predictor variables only accounted for 34% or 22.7% of the variance (Wheaton et al., 2012; Faramarzi et al., 2014). In this article, we also aim to use machine learning to predict the nonlinear relationship between independent and dependent variables in the prediction of psychological status.

Machine learning can deal with big data in high velocity and a variety of forms, so it has been widely implemented in accurately predicting mental health problems, such as anxiety, depression, obsessive-compulsive disorder (OCD), and post-traumatic stress disorder (PTSD) (Kumar et al., 2020; Silveira et al., 2020; Tennenhouse et al., 2020; Xing et al., 2020); classification or diagnosis (Peng et al., 2013; Thabtah, 2018); predicting self-harm and imputing its presence as a missing phenotype (Kumar et al., 2020); and also in distinguishing patients with bipolar disorder from healthy individuals with neuroimaging (Mwangi et al., 2016), neurocognitive data (Wu et al., 2016, 2017a,b), and serum biomarkers (Pinto et al., 2017). This technique includes pattern recognition through the use of complex computational algorithms fed by large data and has the potential to create a paradigm shift in the prediction and stratification of clinical outcomes (Passos et al., 2016; Librenza-Garcia et al., 2017; Silveira et al., 2020). As reported by Ge et al. (2020), machine learning can be used in predicting later clinical outcomes by combining multiple pieces of information from different domains in an effective way and allowing the identification of the most predictive combination of domains. Compared to the traditional statistical prediction models, machine learning approaches may also have advantages in accounting for complex relationships between variables that may not have been previously identified and preventing potentially inaccurate model specifications (Tennenhouse et al., 2020). When there are larger and more complex variables, machine learning becomes a useful technique to disentangle variables associated with outcomes (Iniesta et al., 2016; Dwyer et al., 2018).

This article aims to investigate the prevalence and severity of anxiety among Chinese non-graduating college students and compare the difference between the anxiety status before and 1 month after the start of the new semester of online learning during COVID-19. We also test the ability of the XGBoost model to predict the anxiety level and change of anxiety level 4 weeks after the start of new semester based on the student scores we collected at the start of the new semester. This study is the first to compare the anxiety level of college students before and after the start of a new semester during the COVID-19 pandemic. Our hypothesis is that more non-graduating college students will have a higher level of anxiety in the new semester during COVID-19.

## Materials and Methods

### Participants

A total of 3800 non-graduating college students aged between 18 and 22 from a top multidisciplinary and research-oriented university directly under the jurisdiction of the Ministry of Education in North China were invited to attend two studies during February 15 to March 17, 2020. In total, 1172 students (female: male = 1.56:1) responded to both studies and the response rate was 30.84% (3611 students attended the first study, however, only 1172 participated in the second one). The students were from 26 colleges and schools within the university which were then categorized into arts or sciences institutions. The participants came from 34 provincial-level administrative units and 260 cities in China, which also represented the distribution of the enrolled students from different regions of China at this university. There were also 36 students from Hubei province and one from Wuhan city, where the majority of cases with COVID-19 were identified during the pandemic in China.

### Measures

The questionnaire package used in these two studies consisted of three components: a sociodemographic questionnaire that required each student to provide their gender, year of study, city or province they were living in, major and colleges or schools; a measure of student anxiety (the Self-Rating Anxiety Scale); and an open question about their most recent concerns.

### The Self-Rating Anxiety Scale (Zung, 1971)

The Self-Rating Anxiety Scale (SAS), developed by Zung (1971), was used to assess the subjective feeling of anxiety in the two studies. The SAS is a 20-item self-report assessment device built to measure anxiety levels. Each question is scored on a Likert-type scale of 1–4 (based on these replies: “a little of the time,” “some of the time,” “a good part of the time,” and “most of the time”). Some questions were negatively worded to avoid the problem of set response. The overall assessment was calculated by the total score. Among the 20 items, 5 were reverse scored. The total raw scores ranged from 20 to 80 and then needed to be converted to an “Anxiety Index” score which was equal to 1.25 × raw score and became the standard score which was then used to determine the clinical interpretation of the level of anxiety. The validity and reliability of the instrument has been found to be adequate among Chinese participants. According to the research on the 1158 participants, the levels of anxiety were classified as 25–49 = normal range; 50–59 = mild anxiety levels; 60–69 = moderate anxiety levels; and 70 and above as severe anxiety levels. The mean and standard deviation (SD) of the participants in the two studies were calculated and listed with the number of participants in each group in Table 1.

**TABLE 1:** *T*-test in the first and second tests.

		**1st test mean (SD) subtotal**	**2nd test mean (SD) subtotal**	***T*-test**	***P-*value**
Total	Subtotal	40.39 (9.98) 1172	40.77 (10.51) 1172	−11.829054	0.000*
	Anxious	58.45 (7.81) 184	57.68 (7.58) 221	2.2251471	0.034*
Males	Subtotal	40.10 (10.88) 458	40.84 (11.28) 458	−2.5731238	0.015*
	Anxious	59.98 (8.93) 74	58.09 (8.81) 96	0.53502706	0.345
Females	Subtotal	40.57 (9.36) 714	40.73 (10.00) 714	−5.500983	0.000*
	Anxious	57.42 (6.81) 110	57.37 (7.14) 125	0.07180205	0.397
Arts	Subtotal	40.73 (9.82) 581	41.25 (10.17) 581	−10.136612	0.000*
	Anxious	58.46 (7.63) 90	57.81 (7.00) 108	0.5537752	0.342
Sciences	Subtotal	40.05 (10.13) 591	40.31 (10.83) 591	−4.5404517	0.000*
	Anxious	58.44 (8.03) 94	57.56 (8.16) 113	0.53794686	0.345
Freshmen		40.07 (09.91) 729	40.44 (10.30) 729	−9.71069	0.000*
	Males	39.95 (10.98) 276	40.48 (11.09) 276	−1.39987	0.150
	Females	40.14 (09.20) 453	40.42 (09.79) 453	−3.65373	0.001*
	Arts	40.79 (10.17) 381	40.89 (10.04) 38111111	−0.66332	0.320
	Sciences	39.29 (09.56) 348	39.95 (10.56) 348	−4.3897	0.000*
Sophomore		40.77 (09.92) 312	41.42 (10.89) 312	−3.60757	0.001*
	Males	40.26 (10.94) 125	40.48 (11.81) 125	−0.13441	0.395
	Females	41.12 (09.18) 187	41.36 (10.26) 187	−0.64179	0.325
	Arts	40.75 (09.01) 157	41.89 (10.30) 157	−2.15881	0.039*
	Sciences	40.80 (10.79) 155	40.95 (11.47) 155	−0.1488	0.394
Junior		41.22 (10.51) 131	41.09 (10.81) 131	0.124503	0.396
	Males	40.46 (10.39) 57	40.48 (11.09) 57	−0.00369	0.399
	Females	41.81 (10.64) 74	41.06 (10.66) 74	0.222458	0.389
	Arts	40.15 (09.63) 43	42.09 (10.90) 43	−0.29124	0.382
	Sciences	41.75 (10.93) 88	40.60 (10.79) 88	0.4133	0.366
Those anxious	Freshmen	58.30 (7.94) 112	57.35 (7.04) 131	1.113449	0.214
	Sophomore	58.23 (8.04) 50	57.64 (7.96) 70	0.122947	0.395
	Junior	59.72 (6.80) 22	60.00 (9.63) 20	−0.00658	0.396

*P < 0.05.

### Procedure

This research was registered and approved by the related ethical committee at the university. The non-graduating college students were invited to participate in the two studies, respectively, during February 15 to 17, 2020, right before the new semester started and March 15 to 17, 2020, 1 month after the new semester based on online learning started. The content of the two studies were the same. Those who agreed in writing to participate were each given an online questionnaire package to complete and return to the researchers.

### Statistical Analysis

Statistical analysis was performed using the SPSS 22.0 software. The participating students were divided into different groups according to their SAS scores. Measurement data were expressed as mean and SD. Counting data were expressed by the number of people (%). The descriptive statistics were conducted to illustrate the demographic and other selected characteristics of the participants. The analysis of the relationship between sex, major, grade, region, and anxiety initially used the two-sample *t*-test. The correlation between the SAS scores and confirmed affected cases in different regions were analyzed by Pearson’s product-moment correlation analysis, and *P* < 0.05 on double sides was statistically significant.

We also used XGBoost (Chen and Guestrin, 2016), a scalable machine learning system for tree boosting, to establish the prediction model of student anxiety. XGBoost is a tree ensemble model using K additive functions to predict the output. The base classifier of XGBoost are classification and regression trees (CART). The trees are learned by defining an objective function and optimizing it. The objective function is determined by the following equation: $O\,b\,j=\sum_{i=1}^{n}l\,\left(y_{i},{\hat{y}}_{i}^{\left(t\right)}\right)+\sum_{i}^{t}\Omega \,\left(f_{i}\right)$. It contains a training loss and a regularization. In our model, there were 20 items in SAS and three variables: gender, major, and grade. We also used stepwise multiple regression to establish the prediction model, and compared the prediction performance.

## Results

### Overall SAS Mean Scores in the Two Tests

The results demonstrated in Table 1 show that the mean SAS score in the second study was significantly higher than in the first study (*P* ≤ 0.001). There were 184 (15.7%, Mean = 58.45, SD = 7.81) and 221 (18.86%, Mean = 57.68, SD = 7.58) students who met the cut-off of 50 and were screened as positive for anxiety, respectively, in the two investigations. Both males (*P* < 0.05) and females (*P* ≤ 0.001) had a significantly higher level of anxiety in the second study, however, there were no differences on the level of anxiety for those who were identified as anxious in the two studies (*P* > 0.05). It was also found that students majoring in arts and sciences had a significantly higher level of anxiety in the second study than in the first study (*P* ≤ 0.001), but there were not any statistically significant differences among those who were identified as anxious (*P* > 0.05), though the numbers of participants who were screened as positive in the second study were more than in the first study.

### Group Comparisons in the Two Tests

Table 1 also shows the comparison of the SAS mean scores in different grades in the two studies. Among freshmen, their mean SAS scores were significantly higher in the second study than in the first study (*P* ≤ 0.001), and it was also true in females (*P* = 0.001) and those majoring in sciences (*P* ≤ 0.001) but not in males (*P* = 0.15) and those majoring in arts (*P* = 0.32). Among sophomore students, their mean SAS scores were significantly higher in the second study than in the first study (*P* = 0.001), and also among those majoring in arts (*P* < 0.05) but not in males (*P* = 0.395), females (*P* = 0.325), and those majoring in sciences (*P* = 0.394). Among junior students, there were no significant differences in the mean SAS scores between the two studies (*P* > 0.05). For those who were identified as anxious in both studies, there were no statistically significant differences in the SAS mean scores among participants in each grade (*P* > 0.05).

### SAS Score Ranges in the Two Tests

The SAS score ranges of the participants in the two tests were also calculated. There were 184 (15.7%) and 221 (18.86%) students who met the cut-off of 50 and had different levels of anxiety, respectively, in the two studies. Such as, there were more students identified with mild anxiety in the second study (*N* = 151, 12.88%) than in the first study (*N* = 117, 9.98%); roughly the same number of students with moderate anxiety; but more students with severe anxiety in the second study (*N* = 18, 1.54%) than the first one (*N* = 13, 1.11%). A total of 109 students (9.30%) were identified as anxious in both studies. Among male students, there were 74 (16.16%) in the first test and 96 (20.96%) in the second test that met the cut-off of 50; and among females, 110 (15.41%) and 125 (17.51%) were identified as anxious, respectively, in the two tests, which showed an increase of males and females who were anxious 1 month after the start of the new semester. Similar results were also found among students who were majoring in arts and sciences, among freshmen and sophomore, but not junior students.

As mentioned in *Participants*, there were 36 students from Hubei province, among whom one was from Wuhan city. The student from Wuhan city was screened as positive for anxiety in the second study (SAS = 51.25) but not in the first study (SAS = 40). Among all the 36 students from Hubei province, where the majority of affected cases of COVID-19 were confirmed in China, eight students (22.22%) had SAS scores higher than 50 (6 at the mild and 2 at the moderate level) in the first study and 12 students (33.33%) met the cut-off of 50 (10 at the mild, 1 at the moderate, and 1 at the severe level) in the second study. Seven students (19.44%) were identified as anxious in both studies and one had a moderate level of anxiety in the first study but was normal in the second study. Fourteen students (38.89%) had higher SAS scores in the second study than in the first study.

### SAS Scores of Participants Who Were Identified as Anxious in the Two Tests

Table 2 demonstrates the SAS mean scores and numbers of participants who were identified as anxious. The SAS mean scores were significantly lower in the second study (Mean = 57.68, SD = 7.58) than in the first study (Mean = 58.45, SD = 7.81) (*P* < 0.05), though the numbers of anxious participants in the second study (*N* = 221) were more than in the first study (*N* = 184). There were more males, females, and students majoring in arts and sciences who met the cut-off of anxiety in the second study than in the first study, though their mean SAS scores, respectively, were not significantly different in the two studies (*P* > 0.05). As shown in Table 2, the majority of participants (*N* = 729, 62.2%) were freshmen. The number of sophomore and junior students who were anxious were 312 (26.62%) and 131 (11.18%) respectively, however, there were no significant differences found in the SAS mean scores among the anxious participants in each grade (*P* > 0.05). In both the first and second academic year, there were more students identified as anxious in the second study than in the first study, but roughly the same numbers of anxious participants among junior students.

**TABLE 2:** Self-Rating Anxiety Scale (SAS) mean scores of participants who were identified as anxious in the two tests (number, mean, and SD).

**Mean (SD)**	**Grade**	**Freshmen**	**Sophomore**	**Junior**	**Total**
Total	1st subtotal	112	50	22	184
	1st	58.30 (7.94)	58.23 (8.04)	59.72 (6.80)	58.45 (7.81)
	2nd subtotal	131	70	20	221
	2nd	57.35 (7.04)	57.64 (7.96)	60.00 (9.63)	57.68 (7.58)
Males	1st subtotal	44	20	10	74
	1st	60.17 (9.03)	60.50 (9.94)	58.13 (6.67)	59.98 (8.93)
	2nd subtotal	57	29	10	96
	2nd	57.63 (7.59)	58.58 (9.17)	59.25 (8.82)	58.09 (8.81)
Females	1st subtotal	68	30	12	110
	1st	57.10 (6.95)	56.71 (6.21)	61.04 (6.91)	57.42 (6.81)
	2st subtotal	74	41	10	125
	2nd	57.13 (6.59)	56.98 (7.02)	60.75 (10.80)	57.37 (7.14)
Arts	1st subtotal	64	22	4	90
	1st	58.75 (8.12)	57.05 (6.23)	61.56 (6.32)	58.46 (7.63)
	2st subtotal	65	35	8	108
	2nd	58.00 (6.97)	57.18 (6.23)	59.06 (7.90)	57.81 (7.00)
Sciences	1st subtotal	48	28	18	94
	1st	57.71 (7.74)	59.15 (9.22)	59.31 (7.01)	58.44 (8.03)
	2st subtotal	66	35	12	113
	2nd	56.70 (7.10)	58.11 (8.89)	60.63 (10.93)	57.56 (8.16)

### XGBoost Prediction Model

Scikit-learn, also known as sklearn, is an open source library for machine learning based on Python that supports four machine learning algorithms: classification, regression, reduction, and clustering. We applied the XGBClassifier function of the XGBoost module in the sklearn library.

In this prediction model, the features of participants in the first test can forecast the anxiety levels (normal, mild, moderate, and severe) and changes of anxiety levels (increased, decreased, and unchanged) in the second test. We ranked predictive variables in the model by applying the plot_importance function in the XGBoost module. The feature importance is calculated by gain. The importance of the 20 items in the SAS in the first and second prediction models were both above 95%. So we built two XGBoost classifier prediction models. In one model, we used the 20 items of SAS in the first test together with gender, major, and grade (23 variables altogether) as the feature matrix (X) and in the other model, we only used the 20 items of SAS in the first test as the feature matrix (X). The anxiety levels in the second test and the changes of the anxiety levels were, respectively, used as the labels (y) to train the model and make the prediction. The training set and test set were divided on a scale of 7:3. We adjusted the parameters to construct the best model. We set XGBoost to do multiclass classification using the softmax objective and respectively, set num_class to 4 and 3. We specified the evaluation metrics as merror which was the multiclass classification error rate. The parameter settings are shown in Table 3, and all other parameters that are not in the table were the default values.

**TABLE 3:** Adjusted parameters.

**Parameter**	**Meaning**	**Value**
n_estimators	Number of boosting rounds	1000
max_depth	Maximum tree depth for base learners	8
learning_rate	Boosting learning rate	0.1
Objective	The learning task and the corresponding learning objective or a custom objective function to be used	multi:softmax
Subsample	Subsample ratio of the training instance	0.8
colsample_bytree	Subsample ratio of columns when constructing each tree	0.8
early_stopping_rounds	Activates early stopping	10
eval_metric	Evaluation metrics for validation data	merror

The XGBoost model prediction results are shown in Table 4. The accuracy rate was approximately 80%, an ideal result. Therefore, the anxiety levels of the participants can be accurately predicted and it can be possible to implement effective measures before the anxiety levels increase.

**TABLE 4:** XGBoost prediction results.

**Models**	**Prediction methods**	**Accuracy rate**
Model 1 (23 items)	Anxiety levels (normal, mild, moderate, and severe)	83.81%
	Changes of anxiety level (increased, decreased, and unchanged)	79.26%
Model 2 (20 items of SAS)	Anxiety levels (normal, mild, moderate, and severe)	82.10%
	Changes of anxiety level (increased, decreased, and unchanged)	84.38%

We also conducted multiple linear stepwise regression analysis. The prediction results of multiple linear stepwise regression on the anxiety levels (normal, mild, moderate, and severe) are demonstrated in Table 5 and the prediction results of multiple linear stepwise regression on the changes of anxiety levels (increased, decreased, and unchanged) are shown in Table 6. Table 6 shows that there was linear association between the items listed in the table, Nos. 11, 6, 19, 4, 14, 9, 16, 17, 10, 20, 15, and 18 in the first test and the anxiety levels in the second test (*P* < 0.05). Among these items, the level of No. 6 “My arms and legs shake and tremble” affected the anxiety level in the second test most, which was 12%. Besides, the explanation rate of the regression equation to the anxiety level in the second test was 27.6% (*R*^2^ = 0.28, *R*^2^_*adj*_ = 0.276).

**TABLE 5:** The prediction results of multiple linear stepwise regression on the anxiety levels (normal, mild, moderate, and severe).

**Factors**	**β**	**S_*x*_**	**β′**	***t***	***P***
Items	0.776	0.118		6.577	0.000
11. I am bothered by dizzy spells	0.103	0.033	0.095	3.103	0.002
6. My arms and legs shake and tremble	0.189	0.046	0.120	4.134	0.000
19. I fall asleep easily and get a good night’s rest	−0.062	0.018	−0.097	−3.476	0.001
4. I feel like I’m falling apart and going to pieces	0.070	0.028	0.075	2.520	0.012
14. I get feelings of numbness and tingling in my fingers and toes	0.192	0.051	0.114	3.746	0.000
9. I feel calm and can sit still easily	−0.056	0.019	−0.085	−2.993	0.003
16. I have to empty my bladder often	0.076	0.024	0.084	3.132	0.002
17. My hands are usually dry and warm	−0.053	0.017	−0.085	−3.191	0.001
10. I can feel my heart beating fast	0.095	0.030	0.093	3.141	0.002
20. I have nightmares	0.065	0.025	0.072	2.605	0.009
15. I am bothered by stomach aches or indigestion	0.062	0.026	0.067	2.420	0.016
18. My face gets hot and blushes	−0.076	0.032	−0.067	−2.346	0.019

**TABLE 6:** The prediction results of multiple linear stepwise regression on the changes of anxiety levels (increased, decreased, and unchanged).

**Factors**	**β**	**S_*x*_**	**β′**	***t***	***P***
Items	2.196	0.107		20.489	0.000
8. I feel weak and get tired easily	−0.103	0.022	−0.147	−4.692	0.000
4. I feel like I’m falling apart and going to pieces	−0.121	0.027	−0.132	−4.410	0.000
18. My face gets hot and blushes	−0.159	0.030	−0.143	−5.265	0.000
2. I feel afraid for no reason at all	−0.058	0.027	−0.076	−2.140	0.033
13. I can breath in and out easily	0.048	0.012	0.098	3.940	0.000
11. I am bothered by dizzy spells	−0.130	0.032	−0.123	−4.045	0.000
5. I feel that everything is all right and nothing bad will happen	0.049	0.019	0.073	2.526	0.012
14. I get feelings of numbness and tingling in my fingers and toes	0.137	0.046	0.083	2.988	0.003
7. I am bothered by headaches, neck, and back pains	−0.053	0.023	−0.069	−2.310	0.021
3. I get upset easily or feel panicky	−0.057	0.026	−0.079	−2.187	0.029
17. My hands are usually dry and warm	0.034	0.016	0.056	2.160	0.031

Table 6 also shows that there was linear association between the items listed in the table, Nos. 8, 4, 18, 2, 13, 11, 5, 14, 7, 3, and 17 in the first test and the anxiety levels in the second test (*P* < 0.05). Among these items, the level of No. 8 “I feel weak and get tired easily” affected the anxiety level in the second test most, which was −14.7%. Besides, the explanation rate of the regression equation to the anxiety level in the second test was 32.1% (*R*^2^ = 0.327, *R*^2^_*adj*_ = 0.321).

## Discussion

### Overall Anxiety Is Higher 1 Month After the Start of the New Semester of Online Learning

Consistent with our hypothesis, the non-graduating undergraduate students had an overall higher level of anxiety and more students were identified as anxious 1 month after the new semester based on online learning started, which was also true among each group such as males, females, and students majoring in arts and sciences. College students are at the early stage of adulthood, lack analytical and decisive abilities and experiences, have unstable emotions and hence are inclined to have impulsive behaviors and be affected by public emergencies (Tan, 2003; Taylor, 2006; Li, 2007; Mei et al., 2011).

In China, the pandemic was first detected in December 2019, reached its peak in mid-February, and then from mid-March when the daily news confirmed that patients reached almost zero, the whole COVID-19 situation was under control (Chinese Center for Disease Control and Prevention, 2020). Many previous studies have focused on the initial stage or more general psychological states during the outbreak, while how anxiety levels and severity changed during this time were still unknown. In this article, we collected data on the psychological status of college students in mid-February when COVID-19 was most prevalent and 4 weeks later in mid-March when the pandemic was stable and under control. At the time of the second test, COVID-19 was assumed to have less of an impact on students. But their anxiety level became higher. This may be due to school closure, social distancing or isolation, and online learning. For college students, especially, a lack of social activities and peer interaction, prolonged holidays, and confounded academic planning, etc., would all account for higher risks of anxiety, fear, stress, and depression (Chang et al., 2020). Unlike China, the pandemic began to boost around early March in other countries and newly confirmed cases were still increasing dramatically in mid-July (World Health Organization, 2020). It would be interesting to compare the differences of psychological consequences on college students before and after the start of a new semester based on online learning in China and the rest of the world where the pandemic was still prevalent.

### Group Comparisons

Consistent with previous studies, the younger college students (freshmen and sophomore) had an increased level of anxiety in the new semester but not among junior students as the older the students the more experience they have, and hence their better social adaptive abilities and psychological resilience. It was also proposed in previous research that more prevention measures should be taken to protect the mental health of young students in universities (Yi et al., 2010; Chang et al., 2020). The findings on males and females also confirmed findings from previous studies that females were more vulnerable and more easily affected psychologically. Therefore, female students were found to have a higher level of anxiety in the second test than in the first test, but not among males. Students majoring in arts and sciences both showed higher anxiety in the new semester. However, no differences in the anxiety level of students majoring in arts or sciences between the two tests were found among junior students, which confirmed the findings about the differences of students in the junior grades or more senior grades.

### Correlation of SAS Scores and Confirmed Affected Cases or Regions

Different from a previous study that found no correlation between SAS scores and confirmed affected cases (Wang and Zhao, 2020), this study showed that students from Hubei province, where most cases of COVID-19 were confirmed, had a higher percentage of participants with anxiety. This could be explained by the fact that in the new semester the pandemic had a prolonged influence on college students, even though the newly confirmed cases in each city or province had been close to zero. This gave us a hint that even if their anxiety level was not significantly high during the outbreak, the impact of COVID-19 on the psychological states of college students would remain high for quite a while, therefore, measures should be taken to protect and prevent.

### XGBoost Prediction Model

Comparing the two XGBoost models, model 1 performed better on the prediction of anxiety level with an accuracy rate of 79.26%, however, model 2 had higher prediction accuracy on the changes of anxiety levels (84.38%). It demonstrated that variables such as gender, grade, and major improved the prediction accuracy on anxiety level but not on the changes of anxiety levels. The results also showed that the performance of the multiple linear regression models was much lower than that of the XGBoost prediction model, as the former could only explain 27.6% of the anxiety level in the second test and 32.1% of the change of anxiety levels in the second test. Hence this article successfully tested the feasibility of the XGBoost model in predicting anxiety level and change of anxiety level in the new semester (Chen and Guestrin, 2016).

### Limitations of This Study

This research has several limitations. Firstly, our sample was small and would find it hard to reflect the actual pattern of general non-graduating undergraduate students, given the limited resources available and time-sensitivity of the coronavirus outbreak. The response rate in the first study was 99.86%, however, the majority of the students were not interested in participating in the same test for a second time. In future studies, we propose that we need to make it clear when starting the first test that the research consists of two parts and that one will take place 1 month later to increase the faithfulness of students to this study. Secondly, the self-reported levels of psychological impact, such as anxiety, may not always be consistent with the assessments of professionals. Thirdly, due to the length requirement, we did not collect information on whether the participants had family members who were suspected of or had confirmed cases of COVID-19 which could affect their level of anxiety to a great degree, independent of the time of the tests.

## Conclusion

It was concluded that Chinese non-graduating undergraduate students showed higher anxiety in the new semester based on online learning than before the new semester started during the COVID-19 pandemic. More students from Hubei province had different levels of anxiety than from other provinces. Families, universities, and society should pay attention to the psychological health of non-graduating undergraduate students and take measures accordingly. In addition, as this research was the first to compare the impact of COVID-19 on the anxiety of undergraduate students before and after the start of a new semester based on online learning, this study provides invaluable information on the initial psychological anxiety among university students during the early stage of the COVID-19 pandemic from participants across 260 cities in China and the data could also be used as a baseline to further explore the changes and causes of and strategies to reduce their anxiety.

Besides, this article applied XGBoost models to successfully predict the anxiety level and the changes of anxiety levels 4 weeks later based on their SAS scores in the first test. It also confirmed that the XGBoost model had better prediction accuracy compared to the traditional multiple stepwise regression model on the anxiety status of university students.

This research demonstrated the potential of traditional statistical and machine learning models for identifying predictors of anxiety disorder in college students, and has provided insight into which items are most predictive. Areas for future work include external validation of prediction model results, exploration of the predictive ability of the top items for each instrument separately, and subgroup analyses in external datasets with larger and more complex sample sizes, to further assess machine learning model performance among individuals with anxiety conditions. And this XGBoost model could also be implemented in contexts like the global pandemic and the new needs institutions have to address.

## Data Availability Statement

The raw data supporting the conclusions of this article will be made available by the authors, without undue reservation.

## Ethics Statement

The studies involving human participants were reviewed and approved by the Qiang Li, Chairman of Nankai University, Department of Social Psychology Ethics Committee. The patients/participants provided their written informed consent to participate in this study.

## Author Contributions

CW and HZo co-designed the study. HZo conducted the study and analyzed the data. CW interpreted the data, and wrote and revised the manuscript. HZn helped with the data analysis of XGBoost. All authors contributed to the article and approved the submitted version.

## Conflict of Interest

The authors declare that the research was conducted in the absence of any commercial or financial relationships that could be construed as a potential conflict of interest.
